# LncRNA FGD5-AS1 promotes tumor growth by regulating MCL1 via sponging miR-153-3p in oral cancer

**DOI:** 10.18632/aging.103476

**Published:** 2020-07-16

**Authors:** Chao Ge, Jiali Dong, Yahui Chu, Sumin Cao, Jing Zhang, Jianming Wei

**Affiliations:** 1 Department of Stomatology Clinic, Cangzhou Central Hospital, Cangzhou, Hebei Province, China; 2 Department of Laboratory Medicine, Yi He Maternity Hospital, Cangzhou People's Hospital, Cangzhou, Hebei Province, China

**Keywords:** oral cancer, lncRNA FGD5-AS1, miR-153-3p, MCL1, tumor growth

## Abstract

Purpose: To investigate the function of long noncoding RNA (lncRNA) FGD5-AS1 in oral cancer (OC) and to further clarify the regulation of FGD5-AS1 on miR-153-3p/MCL1 axis.

Results: FGD5-AS1 was significantly increased in OC tissues and cells. Loss of FGD5-AS1 inhibited the proliferation, migration and invasion of OC cells. FGD5-AS1 acted as a sponge of miR-153-3p, and MCL1 was direct target of miR-153-3p. Forced expression of miR-153-3p or inhibition of MCL1 reversed the promoted role of FGD5-AS1 on OC cells’ migration and invasion. The in vivo tumor growth assay showed that FGD5-AS1 promoted OC tumorigenesis by regulating miR-153-3p/MCL1 axis.

Conclusions: Our research revealed lncRNA FGD5-AS1 acted as an oncogene by regulating MCL1 via sponging miR-153-3p, thus providing some novel experimental basis for clinical treatment or prevention of OC.

Patients and Methods: The mRNA expression of FGD5-AS1, miR-153-3p and MCL1 was detected by qRT-PCR. CCK8 assay, Edu assay, wound healing assay and transwell assay were used to detect the FGD5-AS1/ miR-153-3p/MCL1 axis function on proliferation, migration and invasion in OC cells. Western blot was used to calculate protein level of MCL1. Luciferase assay was used to detect the binding of miR-153-3p and MCL1, FGD5-AS1and miR-153-3p.

## INTRODUCTION

Oral cancer (OC) is the third most common cancer in developing countries and the sixth most common cancer worldwide [1]. At present, surgery combined with radiotherapy and chemotherapy has been widely used in the treatment of OC, but the morbidity and mortality of OC are still high [2]. If detected early, the long-term prognosis of patients can be greatly improved [3]. However, OC is prone to regional and distant metastasis, and the size and infiltration of the tumor affect the survival rate of patients. Moreover, once lymph node metastasis occurs, the prognosis is extremely poor, and many patients are already in the advanced stage when they seek medical treatment [4]. Therefore, the monitoring and early detection of OC are very important. Finding appropriate OC tumor markers is of great clinical value in identifying the mechanism of OC occurrence and metastasis and finding new treatment methods.

According to the whole genome sequencing results, in the human genome about 93% of the sequences can be transcribed. Only 1.5% of the transcripts have protein coding function, while non-coding RNA (ncRNA) without protein coding function accounts for 98% of the transcripts [5]. Long non-coding RNA (lncRNA) transcripts are between 200 nt and 100 kt in length [6]. Recent studies have found that a variety of lncRNAs are up-regulated or down-regulated in oral cancer patients, which is closely related to the occurrence, development and prognosis of oral cancer [7]. The study indicated that lncRNA HOTAIR was highly expressed in oral cancer, which was related to the progression oral squamous cell carcinoma (OSCC) [8]. Further experiments in vitro showed that HOTAIR knockdown by siRNA inhibited the proliferation and cloning of OSCC cells. HOTAIR inhibited the expression of e-cadherin by binding the PRC2 complex unit EZH2 and histone H3K27me3 to the promoter of e-cadherin, so it was speculated that the expression of lncRNA HOTAIR was closely related to the occurrence, development and metastasis of OSCC, which might be a sign of poor survival rate of OSCC [9]. LncRNA FGD5-AS1 has been reported to expressed in colorectal cancer and acted as a tumor promoter by sponging miR-302e [10]. What’s more, the expression of FGD5-AS1 was abnormally up-regulated in OSCC tissues and cells. Overexpression of USP21 induced by upregulation of FGD5-AS1 promoted the development of OSCC [11]. Downregulation of FGD5-AS1 inhibited cell growth, migration and invasion, but promoted apoptosis. However, the molecular mechanism of FGD5-AS1 in OC remains unknown.

Myeloid cell leukaemia-1 (MCL1) is a member of the bcl-2 family [12], and many literature researches revealed that MCL1 acted an important part in the survival and death of a variety of cells [13, 14]. In recent years, it has been found that MCL1 is highly expressed in many malignant tumors, and is closely related to the differentiation degree and prognosis of tumors [15]. Reducing the expression of MCL1 can inhibit the proliferation of tumor cells and lead to cell cycle arrest [16]. Therefore, MCL1 has become one of the effective targets for tumor treatment. However, whether FGD5-AS1 regulates MCL1 in OC remains elusive. The purpose of our study was to clarify the specific function of lncRNA FGD5-AS1 in OC to further clarify the regulation of FGD5-AS1 on miR-153-3p/MCL1 axis.

## RESULTS

### Up-regulation of lncRNA FGD5-AS1 in OC tissues and cells

**Figure 1:**
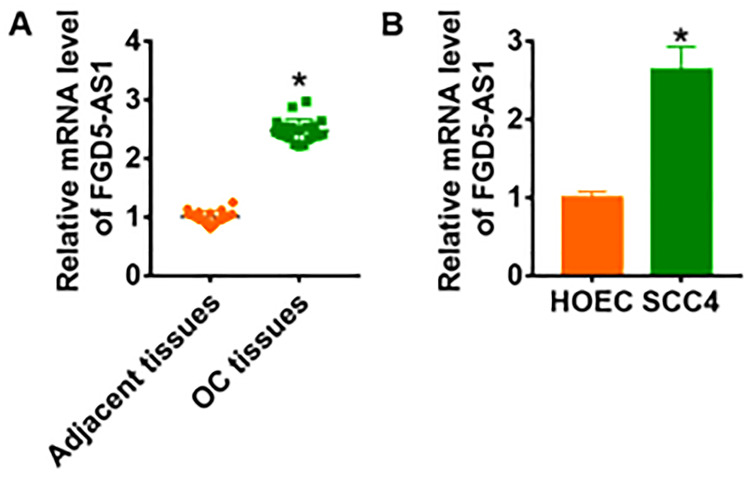
**LncRNA FGD5-AS1 is increased in OC tissue and cells.** (**A**) The expression of FGD5-AS1 in OC tissues (n = 20) and adjacent normal tissues (n = 20) determined by qRT-PCR (*p<0.05). (**B**) qRT-PCR assay analyzed the expression of FGD5-AS1 in human oral epithelial cells (HOEC) and OC cell lines SCC4, SCC-25 and CAL-27 (*p<0.05).

We firstly examined the expression of lncRNA FGD5-AS1 in OC clinical samples. In 20 samples of patients diagnosed with OC, FGD5-AS1 was highly expressed in OC cancer compared with adjacent tissues (Figure 1A). In addition, we cultured OC cell line SCC4 and normal human oral epithelium HOEC. QRT-PCR analysis showed that the level of FGD5-AS1 was dramatically higher in SCC4 than that in HOEC (Figure 1B).

### Silencing lncRNA FGD5-AS1 inhibited proliferation, migration and invasion of OC cells

**Figure 2:**
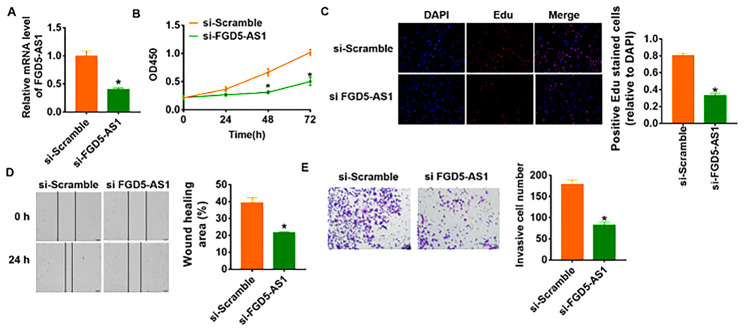
**Knockdown of FGD5-AS1 inhibits proliferation, migration and invasion in SCC4 cells.** (**A**) The expression of FGD5-AS1 in SCC4 cells after FGD5-AS1 si-RNA or si-Scramble transfection was determined by qRT-PCR (*p<0.05). (**B**) CKK-8 assay was used to examine the cell growth at 0, 24, 48 and 72 h (*p<0.05). (**C**) Edu assay was used to calculated cell proliferation (*p<0.05). (**D**) Wound healing assay was used to detect cell migration (*p<0.05). (**E**) Transwell assay was performed to check cell invasive ability (*p<0.05).

To further identify the function of FGD5-AS1 in OC progression, we constructed siRNA of FGD5-AS1. As shown in Figure 2A, transfection of si-FGD5-AS1 significantly suppressed the expression of FGD5-AS1 in SCC4 cells as compared to cells transfected with scrambled siRNA. CCK-8 experiment was performed to detect the proliferation ability in SCC4 after FGD5-AS1 siRNAs transfection. The results showed that loss of FGD5-AS1 significantly inhibited growth rate of SCC4 cells at 48 h and 72 h than cells transfected with scrambled siRNA (Figure 2B). Edu analysis was performed to examine cell proliferative potential, which showed that knockdown of FGD5-AS1 decreased Edu positive cell numbers (Figure 2C). Migration and invasion are the key processes for cancer progression, we then tested the role of FGD5-AS1 on migration and invasion ability in SCC4 cells. The wound healing suggested that silencing FGD5-AS1 decreased the wound healing area, which exhibited a lower migratory ability in si-FGD5-AS1 transfected cells (Figure 2D). Transwell assay was used to test invasive ability. The results showed that si-FGD5-AS1 significantly reduced the cell invasive ability in SCC4 cells (Figure 2E).

### FGD5-AS1 acted as a sponge of miR-153-3p.

**Figure 3:**
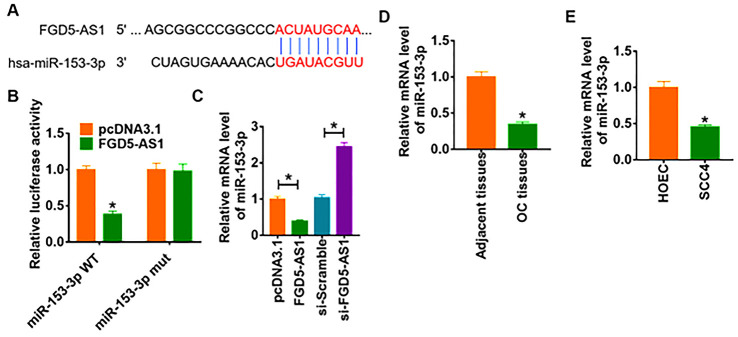
**FGD5-AS1 regulates miR-153-3p expression.** (**A**) The paired bases of FGD5-AS1 with miR-153-3p. (**B**) WT and mutant miR-153-3p luciferase plasmids were transfected into HEK293 cells with pcDNA3.1 or FGD5-AS1. The luciferase activity was measured by dual-luciferase reporter assay system. (*p<0.05). (**C**) FGD5-AS1 or si- FGD5-AS1 or its NC was transfected into SCC4 cells. The mRNA level of miR-153-3p was analyzed by qRT-PCR (*p<0.05). The mRNA level of miR-153-3p in OC tissues (**D**) and cells (**E**) was detected by qRT-PCR (*p<0.05).

We used miRanda database to identify miRNA with FGD5-AS1 biding sites, which showed a binding between miR-153-3p and FGD5-AS1 (Figure 3A). To further clarify the relationship between FGD5-AS1 and miR-153-3p, we performed dual-luciferase reporter assay in HEK293 cell line, and found that the luciferase activity of WT 3’UTR of miR-153-3p was significantly repressed in the FGD5-AS1 compared with pcDNA3.1. However, FGD5-AS1 had no effect on the luciferase activity of mutant miR-153-3p (Figure 3B). Furthermore, qRT-PCR analysis showed overexpression of FGD5-AS1 significantly inhibited miR-153-3p level, while si-FGD5-AS1 promoted miR-153-3p expression (Figure 3C). In addition, we detected miR-153-3p expression in clinical OC tissues and cells. As expected, the protein level of miR-153-3p significantly decreased in OC tissue and SCC4 cells (Figure 3D and 3E).

### Silencing lncRNA FGD5-AS1 inhibited progression of OC cells by regulating miR-153-3p

**Figure 4:**
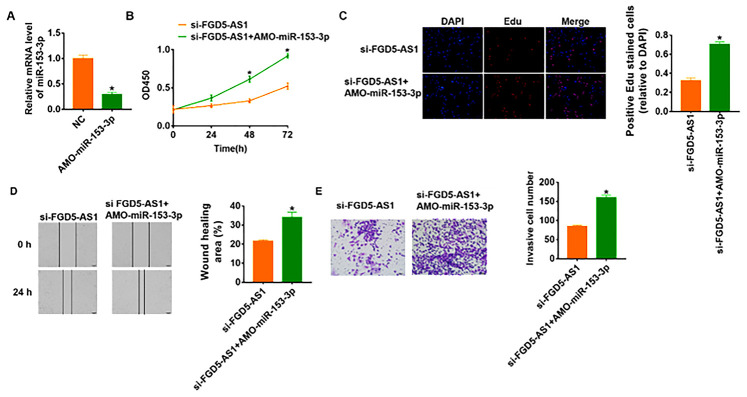
**Knockdown of FGD5-AS1 inhibits OC development by regulating miR-153-3p.** (**A**) The expression of miR-153-3p in SCC4 cells after AMO- miR-153-3p or NC transfection was determined by qRT-PCR (*p<0.05). (**B**) CKK-8 assay was used to examine the cell growth at 0, 24, 48 and 72 h (*p<0.05). (**C**) Edu assay was used to calculated cell proliferation (*p<0.05). (**D**) Wound healing assay was used to detect cell migration (*p<0.05). (**E**) Transwell assay was performed to check cell invasive ability (*p<0.05).

In order to verify whether miR-153-3p is the downstream molecule of FGD5-AS1 regulating OC, we transfected AMO-miR-153-3p in SCC4 cells to inhibit miR-153-3p expression (Figure 4A). And the followed functional experiments showed that loss of miR-153-3p removed the inhibitory effect of FGD5-AS1 on cell viability, proliferation, migration and invasion (Figure 4B–4E), which indicated that si-FGD5-AS1 inhibited OC progression through promoting miR-153-3p.

### MiR-153-3p directly targeted MCL1

**Figure 5:**
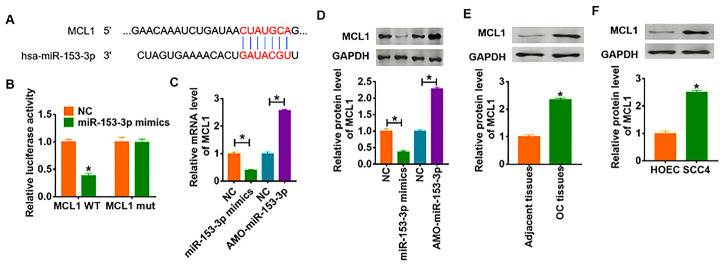
**MCL1 is direct targets of miR-153-3p.** (**A**) Targeting prediction results of miR-153-3p with MCL1. (**B**) WT and mutant MCL1 luciferase plasmids were transfected into HEK293 cells with NC or miR-153-3p. The luciferase activity was measured by dual-luciferase reporter assay system. (*p<0.05). miR-153-3p or AMO-miR-153-3p or its NC was transfected into SCC4 cells. (**C**) The mRNA level of MCL1 was analyzed by qRT-PCR (*p<0.05). (**D**) Western blot was performed to detect MCL1 protein expression of (*p<0.05). The protein level of MCL1 in OC tissues (**E**) and cells (**F**) was detected by western blot (*p<0.05).

To further clarify the downstream mechanism of miR-153-3p regulating OC, we used Targetscan to predict the potential target. And we found that the 3’UTR of MCL1 possessed directed target site for miR-153-3p (Figure 5A). To investigate whether miR-153-3p directly targeted on MCL1, we constructed dual-luciferase reporter plasmid of WT and mutant MCL1. And we found that the luciferase activity of WT 3’UTR of MCL1 was decreased in the miR-153-3p mimic group compared with NC group. However, miR-153-3p had no effect on the luciferase activity of mutant MCL1 (Figure 5B). Furthermore, overexpression of miR-153-3p significantly inhibited both the mRNA and protein expression of MCL1, while AMO-miR-153-3p increased MCL1 level in SCC4 cells (Figure 5C and 5D). What’s more, we detected MCL1 expression in clinical OC tissues and cells, which showed a dramatic increase of MCL1 level in OC tissues and cells (Figure 5E and 5F).

### FGD5-AS1 promoted proliferation, migration and invasion via miR-135-3p/MCL1 axis in OC cells

**Figure 6:**
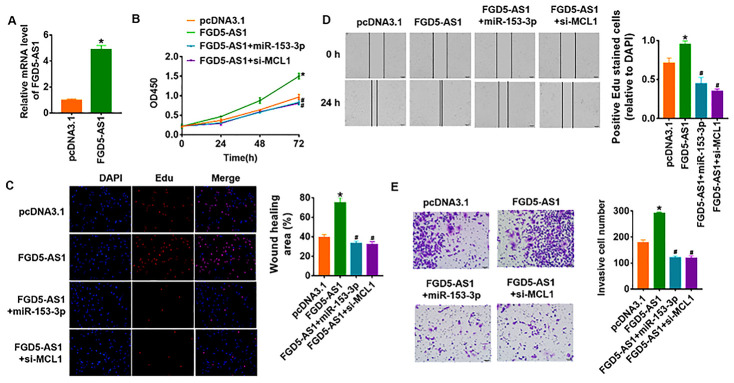
**LncRNA FGD5-AS1 promotes OC progression via miR-153-3p/MCL1 axis.** (**A**) FGD5-AS1 plasmid was transfected into SCC4 cells, and qRT-PCR analysis was used to detect the transfection efficiency (*p<0.05). miR-153-3p or si-MCL1 was cotransfected into SCC4 cells with FGD5-AS1 plasmid. CCK8 assay (**B**) and Edu assay (**C**) was used to calculate cell proliferation (*p<0.05 *vs* pcDNA3.1, ^#^*p*<0.05 *vs* FGD5-AS1). (**D**) Wound healing assay was used to detect cell migration (*p<0.05 *vs* pcDNA3.1, ^#^*p*<0.05 *vs* FGD5-AS1). (**E**) Transwell assay was performed to check cell invasive ability (*p<0.05 *vs* pcDNA3.1, ^#^*p*<0.05 *vs* FGD5-AS1).

To identify whether FGD5-AS1 promotes OC progression via miR-135-3p/MCL1 axis, we forced expression of FGD5-AS1 in SCC4 cells (Figure 6A). As shown in Figure 6B–6E, overexpression of FGD5-AS1 increased cell viability and promoted cell proliferation, migration and invasion in SCC4 cells. Additionally, overexpression of miR-135-3p or knockdown of MCL1 reversed the pro-tumor effect of FGD5-AS1 (Figure 6B–6E). Taken together, FGD5-AS1 regulated the progression of OC via controlling miR-135-3p/MCL1 axis.

### FGD5-AS1 promoted in vivo tumor growth in the nude mice

**Figure 7:**
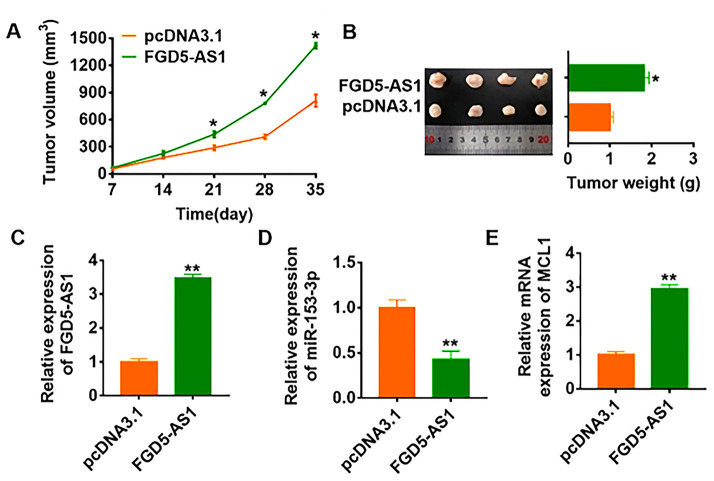
**LncRNA FGD5-AS1 promotes in vivo tumor growth in the nude mice.** The nude mice were subcutaneously injected with SCC4 cells (5 x 10^6^) into the right flanks of the nude mice. 1 week later, we injected lentivirus packaged FGD5-AS1 or pcDNA3.1 into tumors. (**A**) The tumor volume was assessed in the nude mice every 7 days (*p<0.05). (**B**) Tumor weight was determined in the isolated tumors from the nude mice (*p<0.05). (**C**) The relative expression of FGD5-AS1 was determined by qRT-PCR in the isolated tumor tissues (**p<0.01). qRT-PCR was performed to detect the relative mRNA expression of miR-153-3p (**D**) and MCL1 (**E**) (*p<0.05).

To further explore the function of FGD5-AS1 in OC, we set up xenograft nude mice model. 30 mice were divided into two groups randomly, SCC4 cells were subcutaneously injected into nude mice. 1 week later, we injected lentivirus packaged FGD5-AS1 or pcDNA3.1 into tumors, and we measured tumor volume. The mice with FGD5-AS1 showed a bigger tumor volume, and tumors grew faster (Figure 7A). The tumors were isolated at 30 days after injection, FGD5-AS1 significantly increased tumors weight (Figure 7B). In addition, we isolated these tumor tissues and found that FGD5-AS1 was increased in lentivirus packaged FGD5-AS1 injection tumors (Figure 7C). Moreover, FGD5-AS1 decreased the mRNA level of miR-153-3p and increased MCL1 expression (Figure 7D and 7E).

## DISCUSSION

Oral cancer tends to metastasize and has a poor prognosis [17]. OC is a long-term, multi-stage accumulation of genetic changes, involving many genetic and epigenetic changes to alter the expression of oncogenes, tumor suppressor genes and other related molecules [18]. The early detection, diagnosis and treatment of OC have always been the focus of OC research. Searching for effective markers is an important factor to improve the survival and prognosis of oral cancer patients.

At present, many studies on OC focus on miRNAs, while few studies were on ncRNAs [19]. LncRNAs were initially thought to have no biological function and cannot be translated into proteins. But as the expression of chip technology and in situ hybridization technology and depth of the RNA sequencing technology develops, a growing number of studies have found that lncRNA in epigenetic, transcriptional level and the transcription level on multiple levels such as regulation of gene expression [20]. It has been shown that the expressions of miR-26a and lncRNA MEG3 in TSCC were significantly decreased [21]. Further in vitro cell line studies showed that overexpression of miR-26a and MEG3 inhibited proliferation and promoted apoptosis [21].

And in our study, we explored the expression of lncRNA FGD5-AS1 in OC to clarify its function. Surprisingly, FGD5-AS1 was significantly increased in clinical OC tissues, and we also found a remarkable elevation of FGD5-AS1 in OC cell lines. These data indicated that FGD5-AS1 might be involved in the development of OC. To further investigate the role of FGD5-AS1 on OC progression, we constructed siRNA to silence FGD5-AS1 expression. And we found that deficiency of FGD5-AS1 significantly decreased cell viability and inhibited proliferation, migration and invasion of OC cells.

At present, numerous studies have shown that lncRNA participates in a variety of physiological and pathological processes through the ceRNA mechanism [22, 23]. In order to clarify the mechanism of FGD5-AS1 regulating OC, we predicted that a binding between miR-153-3p and FGD5-AS1. FGD5-AS1 was a distinct ceRNA for miR-153-3p. Expression of miR-153-3p was significantly decreased in OC tissues and cells, suggesting meaningful participation of miR-153-3p in OC progression. Further functional experiments showed silencing lncRNA FGD5-AS1 inhibited the ability to proliferate, migrate, and invade in OC cells by regulating miR-153-3p.

MCL1 belongs to Bcl 2 family, and plays an anti-apoptosis role in multiple types of cells [24, 25]. The initiation of normal apoptosis can maintain the balance of the number of cells, while cancer cells can obtain the ability of unlimited proliferation and growth by inhibiting apoptosis through some molecular mechanisms [26, 27]. And in our study, we surprisingly found that MCL1 was a direct target of miR-153-3p. In addition, MCL1 was increased in OC including clinical tissues and cell lines. Furthermore, over expression of miR-153-3p or knockdown of MCL1 reversed the effect of FGD5-AS1 on promoting OC proliferation, migration and invasion. These data suggested that FGD5-AS1 promoted OC progression via miR-153-3p/MCL1 axis. Meanwhile, in vivo tumor formation experiments proved that FGD5-AS1 could significantly promote the progression of OC, which was conducive to clinical targeted therapy.

Revealing the mechanism of the occurrence and development of OC at the molecular level can provide a new and more effective method for the early detection, diagnosis and prognosis of OC. Because OC is difficult to be diagnosed early, easy to metastasize and has a poor prognosis, many studies have been conducted to find more sensitive, specific and valuable tumor markers. The importance of the lncRNA in cancer and OC has obtained more and more attention. Through various research methods such as high flux detection technology, we realized that some lncRNA abnormal expression in OC. And this motivated us into a more in-depth research on lncRNA contact of OC, and we have found some can be used as a monitoring index of OC occurrence and development. So far, the expression pattern of LncRNA and biological functions have been elucidated, part of the many mechanisms is not exact. The next challenge we need to overcome is whether we can effectively control the appropriate expression level of lncRNA in OC. It is believed that with the deepening of research, lncRNA will surely open a new chapter in the diagnosis, monitoring, treatment and prevention of OC.

## CONCLUSIONS

Our study suggested lncRNA FGD5-AS1 acted as an oncogene by regulating MCL1 via sponging miR-153-3p. And FGD5-AS1 might be a potential therapeutic target for OC clinical treatment.

## MATERIALS AND METHODS

### Clinical samples

Fresh cancer tissue samples and adjacent normal tissue samples were taken from 20 oral cancer patients undergoing surgical procedures at Cangzhou Central Hospital. All of the patients or their guardians provided written consent, and the study was approved by the Ethics Committee of Cangzhou Central Hospital.

### Cell culture

The cell lines (SCC4, SCC-25 and CAL-27) were purchased from the Science Cell Laboratory. Cells were cultured in RPMI 1640 (GIBCO, USA) supplemented with 10 % fetal bovine serum (Cromwell, USA) and 100 μL/mL penicillin and streptomycin (Sigma-Aldrich, USA) and placed at 37°C with 5% CO_2_. Cells were transfected with 500 μM miR-153-3p or AMO-miR-153-3p and 2 μg FGD5-AS1 or si-FGD5-AS1 or si-MCL1 plasmid, with Lipofectamine^TM^ 2000 (Invitrogen, Carlsbad, CA, USA).

### RNA isolation and qRT-PCR

RNA isolation, reverse transcription and quantitative expression were carried according to manufacturer’s instructions. All the kits were purchased from Vazyme, and gene expression was calculated using 2^-ΔΔCt^ method.

### Protein isolation and western blot

Total protein was collected from cells with RIPA lysis Mix. Western blotting assay was performed as previously described. Briefly, 60 μg protein extractions were loaded via SDS-PAGE and transferred onto nitrocellulose membranes (absin, China), then incubated with primary antibodies for 2 h at temperature, then plated at 4 °C overnight, the membranes were incubated in 5% non-fat milk blocking buffer in horizontal mode for 3 h. After incubation with secondary antibodies, the membranes were scanned using an Odyssey, and data were analyzed with Odyssey software (LI-COR, USA).

### CCK8 assay

Cells were seeded in 96-well cell plates, and added CCK-8 solution (Vazyme, China) at 0, 24, 48 and 72 h. 2 h later, the OD value was measured at 450 nm.

### Edu assay

Cells were seeded in 24-well cell plates, and added Edu solution (Ribobio, China) according to the protocols. The cells were incubated for 2 h, and then treated and photographed.

### Wound healing assay

5×10^5^ cells were planted in a 6-well plate, and when the cells grew to fuse, two vertical parallel lines were drawn with 10 μL suction head against the ruler. The floating cells were washed with PBS and cultured in serum-free medium for 24 h. Images were taken at 0 and 24 h of cell culture, respectively.

### Transwell assay

Cells in logarithmic growth phase were adjusted to 2 × 10^5^ cells/well of medium (without serum) and plated into the upper chamber insert pre-coated with 1μg/μl Matrigel. Lower chamber was added with 500 μL of medium (with 10% FBS), and then incubated at 37°C for 48 h. Then the invading cells were visualized by the crystal violet and inverted microscope.

### Luciferase assay

psiCHECK-2 luciferase reporter plasmid was inserted with the wildtype MCL1 3’UTR or mutant MCL1 3’UTR sequences that contain the putative binding sites of miR-153-3p. NC or miR-153-3p mimics were transfected with reporter vectors into HEK293 cells. The cells were collected after 48 h post-transfection and lysed to detect the luciferase activity (Promega).

### In vivo tumor growth assay

SCC4 cells (5 x 10^6^) were subcutaneously injected in right lower limb of the nude mice or through abdominal and vail veins. Tumor size was measured every 7 days. After 35 days of injection, mice were intraperitoneally injected with 3% pentobarbital sodium and were killed by excessive anesthesia with a dose of 90 mL/kg, and the tumors were removed for follow-up study.

### Statistical analysis

Significant differences were calculated using two-tailed *t*-test through Graphpad 7.0 and SPSS 22.0.
